# Assessing Pathologic Response in Resected Lung Cancers: Current Standards, Proposal for a Novel Pathologic Response Calculator Tool, and Challenges in Practice

**DOI:** 10.1016/j.jtocrr.2022.100310

**Published:** 2022-03-19

**Authors:** Anjali Saqi, Kevin O. Leslie, Andre L. Moreira, Sylvie Lantuejoul, Catherine Ann Shu, Naiyer A. Rizvi, Joshua R. Sonett, Kosei Tajima, Shawn W. Sun, Barbara J. Gitlitz, Thomas V. Colby

**Affiliations:** a Department of Pathology and Cell Biology, Columbia University Irving Medical Center, New York, New York; b Department of Laboratory Medicine and Pathology, Mayo Clinic Arizona, Scottsdale, Arizona; c Department of Pathology, NYU Grossman School of Medicine, New York, New York; d Department of BioPathology, Léon Bérard Center, Lyon, France; e Department of Pathology, Grenoble Alpes University, Grenoble, France; f Department of Medicine, Columbia University Irving Medical Center, New York, New York; g Herbert Irving Comprehensive Cancer Center, Columbia University, New York, New York; h Department of Thoracic Surgery, NewYork-Presbyterian Columbia University Irving Medical Center, New York, New York; i Biometrics Department, Chugai Pharmaceutical Co., Ltd., Tokyo, Japan; j Product Development Clinical Oncology, Genentech Inc., South San Francisco, California; k Gilead Sciences, Inc., Foster City, California; l Department of Laboratory Medicine and Pathology, Mayo Clinic Arizona, Scottsdale, Arizona

**Keywords:** NSCLC, MPR assessment, pCR, Neoadjuvant therapy, Early-stage lung cancer

## Abstract

The efficacy of neoadjuvant treatment for NSCLC can be pathologically assessed in resected tissue. Major pathologic response (MPR) and pathologic complete response (pCR), defined as less than or equal to 10% and 0% viable tumor cells, respectively, are increasingly being used in NSCLC clinical trials to establish them as surrogate end points for efficacy to shorten time to outcome. Nevertheless, sampling and MPR calculation methods vary between studies. The International Association for the Study of Lung Cancer recently published detailed recommendations for pathologic assessment of NSCLC after neoadjuvant treatment, with methodology being critical. To increase methodological rigor further, we developed a novel MPR calculator tool (MPRCT) for standardized, comprehensive collection of percentages of viable tumor, necrosis, and stroma in the tumor bed. In addition, tumor width and length in the tumor bed are measured and unweighted and weighted MPR averages are calculated, the latter to account for the varying proportions of tumor beds on slides. We propose sampling the entire visible tumor bed for tumors having pCR regardless of size, 100% of tumors less than or equal to 3 cm in diameter, and at least 50% of tumors more than 3 cm. We describe the uses of this tool, including potential formal analyses of MPRCT data to determine the optimum sampling strategy that balances sensitivity against excessive use of resources. Solutions to challenging scenarios in pathologic assessment are proposed. This MPRCT will facilitate standardized, systematic, comprehensive collection of pathologic response data with a standardized methodology to validate studies designed to establish MPR and pCR as surrogate end points of neoadjuvant treatment efficacy.

## Introduction

Lung cancer is the leading cause of cancer deaths worldwide.^1^^,^^2^ NSCLC is often treated systemically, with or without surgical resection, radiotherapy, or both. The objective of systemic therapy for resectable NSCLC is to decrease the risk of local recurrence and metastatic disease. Unlike adjuvant systemic therapy, neoadjuvant therapy allows radiologic or pathologic in vivo monitoring of treatment effect after resection.

Discrepancies with pathologic assessment findings have highlighted challenges in radiologic measurement of response to neoadjuvant therapies.^3^, ^4^, ^5^ Statistically significant correlations between major pathologic response (MPR) and radiographic response, disease-free survival, event-free survival, and overall survival (OS) were revealed by post hoc analyses of neoadjuvant chemoimmunotherapy trials.^6^^,^^7^ In other neoadjuvant chemotherapy and chemoimmunotherapy studies, pathologic complete response (pCR) correlated with partial,^4^ but rarely with complete, radiologic response^8^ on the basis of the Response Evaluation in Solid Tumors, although it was associated with longer 18- and 24-month progression-free survival and OS rates.^8^ Even positron emission tomography or computed tomography^3^ cannot reliably differentiate between residual viable tumor and therapy-related changes, such as fibrosis and macrophages,^9^ within the tumor bed or along its periphery,^10^ conceivably blurring the distinction from bordering non-tumor bed regions. Nevertheless, the available retrospective evidence suggests that pathologic response to neoadjuvant chemotherapy has a potentially stronger correlation with survival outcome than classic radiologic response of the Response Evaluation in Solid Tumors version 1.1.^11^^,^^12^

In 1997, Junker et al.^13^ described substantially longer median survival in patients with NSCLC who had 0% to 10% viable tumor in resections after neoadjuvant chemoradiotherapy than those who had more than 10% viable tumor or no response. Similarly, Pataer et al.^14^ noted significantly greater 5-year OS and disease-free survival in patients with less than or equal to 10% residual viable tumor after neoadjuvant chemotherapy for NSCLC than for patients with more than 10% viable tumor cells. MPR in the lymph nodes was also a reliable predictor of OS in these patients.^15^ These and analogous studies^16^^,^^17^ defined MPR as less than or equal to 10% residual viable tumor and laid the foundation for its potential utilization as a surrogate end point for OS,^18^ the long-established criterion standard of treatment efficacy in NSCLC. As a surrogate end point, MPR has the potential to shorten the time from enrollment to outcome of otherwise decade-long clinical trials and thereby foster innovation in developing new treatments for NSCLC.

In 2020, the International Association for the Study of Lung Cancer (IASLC) published uniform recommendations for pathologic assessment of NSCLC after neoadjuvant therapies, including chemotherapy, radiotherapy, molecular-targeted therapy, and immunotherapy.^10^ The recommendations were formulated to provide guidance because data in this setting for lung cancer are limited, with methodologies for assessing pathologic response described at limited and varying extents in earlier NSCLC studies^4^^,^^5^^,^^11^^,^^13^^,^^14^^,^^19^ (Table 1), in contrast to those in osteosarcoma^20^ and breast cancer.^21^^,^^22^ To address absent or partially described aspects of assessment, the IASLC made recommendations for macroscopic assessment, tumor bed sampling, and microscopic assessment of NSCLC specimens.

**Table 1:** Reported Methodology for Tumor Bed Sampling, Microscopic Assessment, and Definition of Pathologic Response in Prospective and Retrospective Neoadjuvant NSCLC Studies and IASLC Recommendations

Study Aim	No. of Cases	Sampling Methodology	Microscopic Assessment and Definition of Pathologic Response
Prospective clinical studies			
Histologic assessment in a prospective multicenter study after neoadjuvant combined chemotherapy and radiotherapy (Junker et al, 1997)^13^	40 formalin-fixed resection specimens of locally advanced NSCLC	• Samples taken from serial sections of “areas with likely vital tumor growth or previous, now regressively altered, tumor tissue” • “Histologic slides of the surrounding, macroscopically tumor-free parenchymal lung tissue were also prepared”	“To determine the degree of tumor regression, the type and extent of vital tumor tissue and tumor necroses and reactive alterations with foam cell reaction and fibrosis or scar formation were taken into account and correlated to regression grading,” with <10% vital tumor tissue assigned grade IIb, and no evidence of vital tumor tissue assigned grade III, both of which “suggested a good response to neoadjuvant therapy”
Evaluation of induction chemoradiotherapy and surgery in patients with superior sulcus NSCLC (Southwest Oncology Group Trial 9416–Intergroup Trial 0160) (Rusch et al, 2007)^5^	88 patients who underwent surgery; samples not described	Not described (used review of pathology and CT scan reports)	Final pathologic response defined as “pathologic CR (no residual microscopic tumor), minimal microscopic residual (few scattered tumor foci within a mostly necrotic or fibrotic mass), and gross residual disease (mostly or entirely viable tumor)”
Blinded evaluation of ability of histopathologic response to predict outcomes in patients with surgically resected NSCLC treated or not with neoadjuvant chemotherapy (Pataer et al, 2012)^14^	Histologic slides of gross residual tumor from 358 patients	≥1 section per centimeter of greatest tumor diameter (5–30 per patient)	• Percentage of residual tumor estimated by comparing the estimated cross-sectional area of viable tumor foci with estimated cross-sectional areas of necrosis, fibrosis, and inflammation on each slide • Results for all slides were averaged to determine the mean values for each patient
Efficacy of neoadjuvant nivolumab in patients with resectable stages I–IIIA NSCLC (Forde et al, 2018)^23^	21 patients	Resection of primary tumor and lymph nodes completed according to institutional standards	• Primary tumors assessed for the percentage of residual viable tumor • MPR defined as tumors with ≤10% viable tumor cells
Efficacy of neoadjuvant atezolizumab plus chemotherapy in patients with stages IB–IIIA NSCLC (Shu et al, 2020)^6^	30 patients	• Tumor tissue samples were sectioned • Tumor bed samples <6 cm in diameter: submitted in entirety • Tumor bed samples ≥6 cm diameter: ≥1 section per centimeter of greatest diameter assessed for MPR	• Local pathologists measured percentage of residual viable tumor in resected primary tumors at time of surgery • Percentage of viable tumor tissue recorded for each tumor slide before calculating the average percentage of viable tumor tissue for each patient • MPR defined as ≤10% viable residual tumor • In patients who had pCR (defined as absence of viable tumor in all slides), entire tumor bed was examined histologically
Efficacy of neoadjuvant nivolumab plus chemotherapy in patients with stage IIIA resectable NSCLC (NADIM) (Provencio et al, 2020)^8^	41 patients who had surgery	Entire tumor; median, 10 sections (range: 8–28 sections) reviewed for pathologic response	• Local pathologists measured percentage of residual viable tumor in resected primary tumors; confirmed by agreement between two blinded pathologists • MPR defined as ≤10% viable tumor cells in the primary tumor • Incomplete pathologic response: >10% viable tumor cells in the primary tumor • pCR: tumors with no viable tumor cells in the resected lung cancer specimen and all sampled regional lymph nodes
Efficacy of neoadjuvant nivolumab ± ipilimumab in patients with stages I–IIIA resectable NSCLC (NEOSTAR) (Cascone et al, 2021)^11^	37 patients who had surgery on study	• After gross identification of tumor/tumor bed, ≥1 section per centimeter of greatest tumor (bed) diameter submitted for histopathologic evaluation • If no residual tumor identified microscopically, entire tumor bed submitted for review	• After initial clinical reporting, pathologic responses were reviewed by two blinded pathologists experienced in assessing MPR after neoadjuvant therapy, and the average scores were used for final analysis • MPR defined as ≤10% viable tumor cells in the tumor • pCR: 0% viable tumor cells
Efficacy of neoadjuvant nivolumab plus chemotherapy vs. chemotherapy in patients with stages IB–IIIA, *ALK*- and *EGFR*-wild type, resectable NSCLC (CheckMate 816)^31^	284 patients had surgery	Not described in published abstract	• pCR (coprimary end point) was defined as no residual viable tumor in both the resected lung specimen and the sampled lymph nodes after surgery • MPR (secondary end point) was defined as ≤10% viable tumor in the lung and lymph nodes • pCR and MPR were assessed in lung and lymph nodes by blinded independent pathologic review
Efficacy of neoadjuvant durvalumab plus chemotherapy in patients with stage IIIA(N2) NSCLC (SAKK 16/14)^7^	55 patients who had surgery	Not described in manuscript. “Tumor tissue from initial biopsy and resection specimens underwent central pathology review in accordance with WHO classification (fourth edition, 2015) and College of American Pathologists protocol”	• Pathologic response was evaluated by assessing percentage of residual viable tumor volume in relation to tumor bed • MPR (secondary end point) defined as ≤10% viable tumor cells • pCR (secondary end point) defined as no evidence of viable tumor cells
Retrospective studies			
Retrospective analysis in consecutive patients with superior sulcus tumors treated with neoadjuvant chemoradiotherapy (Blaauwgeers et al, 2013)^4^	Tumor material from 46 patients	Not described. “Histologic slides of all available paraffin blocks and the pathology reports of the resection specimens were reviewed, with the number of blocks related to the tumor area being estimated in each case”	• Viable tumor categories were defined and scored per Dworak grading for colorectal cancer (<10% vital tumor cells for squamous cell carcinoma, 10%–15% for adenocarcinoma, and >50% for large-cell NSCLC) • “A continuous area of >6 mm tumor in one slide was considered as sufficient for scoring >10% vital tumor cells” • “A vital tumor cell score <10% was defined as small foci of vital tumor in one or more sections with an estimated total area of less than 10% of the gross size of the lesion”
Retrospective evaluation of whether optimal cutoff percentage of residual viable tumor for predicting survival differed between lung adenocarcinoma and squamous cell carcinoma (Qu et al, 2019)^19^	Tumor slides from 272 patients with stages II–III NSCLC treated with neoadjuvant chemotherapy and surgery	• Median of five sections (range, 1–24 sections) per patient	• Used an Olympus BX51 microscope (Olympus; Tokyo, Japan) with standard 22-mm diameter eyepiece; discrepancies between pathologists resolved using a multihead microscope • Across all tumor sections, percentages of viable tumor area, necrosis, and stroma within the tumor bed were estimated in 5% increments to total 100% of the tumor bed • Viable tumor size was calculated as tumor bed size × percentage viable tumor × (100 – percentage lepidic area)/10,000 • MPR defined as ≤10% viable tumor
Retrospective study to confirm that MPR is predictive of long-term OS in patients with NSCLC after neoadjuvant chemotherapy and surgical resection; assessment of interobserver agreement on MPR between two observers; minimum number of slides needed for accurate determination of tumor response (Weissferdt et al, 2020)^12^	151 patients	• 2–12 slides of paraffin-embedded hematoxylin and eosin–stained tumor sections were examined for each patient • Recommended that initial evaluation should be based on 3 slides ○ If percentage of viable tumor in those slides is consistently >20%, no further slides need to be evaluated ○ If scores are between 5% and 20%, ≥7 slides are required to achieve ≥90% accuracy	• Slides reviewed by an experienced and a new observer, independently then together ○ Levels of agreement between two pathologists were high after direct in-person training (*R*^2^ = 0.994) • Percentage residual tumor was estimated by comparing the estimated cross-sectional area of the viable tumor foci with the estimated cross-sectional areas of fibrosis and necrosis (tumor bed) on each slide • Giant cell reaction, cholesterol cleft granulomas, foamy macrophages, and inflammation were assessed using a score from 0 to 3, and results were averaged from all slides to determine mean value of treatment response for each patient • MPR defined as ≤10% viable tumor cells • MPR was significantly predictive of long-term OS after neoadjuvant chemotherapy on multivariable analysis (HR = 2.68; *p* = 0.01)
Reviews and recommendations			
Review on ideal number of histologic sections that should be evaluated to determine MPR accurately (Oramas and Moran, 2021)^24^	–	• Evaluation of the entire tumor bed is the best method to determine the exact percentages of tumor viability and other nontumoral histopathologic features	• There is a need for an algorithm that incorporates both tumor reduction by imaging and results of histopathologic assessment to provide more accurate information regarding tumor response to therapy
IASLC recommendations (Travis et al, 2020)^10^	–	• Tumor ≤3 cm: Sample entirely • Tumor >3 cm: Take ≈0.5-cm-thick cross-section of tumor at maximum dimension, photograph it, and sample the most representative section revealing viable tumor • Histologic sections at tumor periphery should include tumor border plus ≥1 cm of surrounding non-neoplastic lung parenchyma	• Histologic features of necrosis, stromal tissue, and viable tumor should be estimated to sum 100% of the tumor bed • Informal semiquantitative “eyeball” approach can be used to estimate percentages • Use 10% increments for percentages unless amount is <5% • Calculate MPR as estimated size of viable tumor divided by tumor bed size • MPR: ≤10% viable tumor (cutoff may vary by histologic type) • pCR: 0% viable tumor cells after complete evaluation of resected specimen, including all sampled regional lymph nodes

CR, complete response; CT, computed tomography; HR, hazard ratio; IASLC, International Association for the Study of Lung Cancer; MPR, major pathologic response; No., number; OS, overall survival; pCR, pathologic complete response.

Building on these recommendations and to further aid studies confirming MPR as a surrogate end point of survival, we developed a systematic approach to NSCLC pathologic assessment that is being used in an IASLC interobserver study following patients with NSCLC and to review an estimated 451 lung resections after neoadjuvant chemotherapy with or without checkpoint inhibitor therapy in a phase 3 study (IMpower030; NCT03456063). Here, we propose strategies to increase the rigor of MPR assessment methodology to allow the systematic collection of data, to address unanswered questions in an evidence-based fashion, and to provide a standardized methodology for adoption across clinical trials and potentially in clinical practice. We also propose methods to address challenging morphologic scenarios that may be encountered. Though we discuss reported methodologies for pathologic assessment in prospective and retrospective neoadjuvant NSCLC studies, comparison of the effectiveness of these methodologies is beyond the scope of this work.

## MPR Calculator Tool

Several studies that have evaluated pathologic response in NSCLC described the correlation between the percentage of residual viable tumor and outcomes, but the methods used to calculate the proportion of residual viable tumor have varied (Table 1).^4^^,^^8^^,^^12^^,^^14^^,^^19^^,^^23^ To address this shortcoming, we developed and implemented a novel MPR calculator tool (MPRCT) to facilitate methodical, standardized, comprehensive collection of microscopic data for the tumor bed, which consists of the following three main components: viable tumor, necrosis, and stroma (i.e., fibrosis and inflammation).^10^ When used in conjunction with information in synoptic pathology reports,^10^ radiologic reviews, and clinical outcomes, the MPRCT can be used to reveal evidence-based correlations not previously explored and can thus more concretely and accurately establish pathologic response as a surrogate marker for survival.

**Figure 1:**
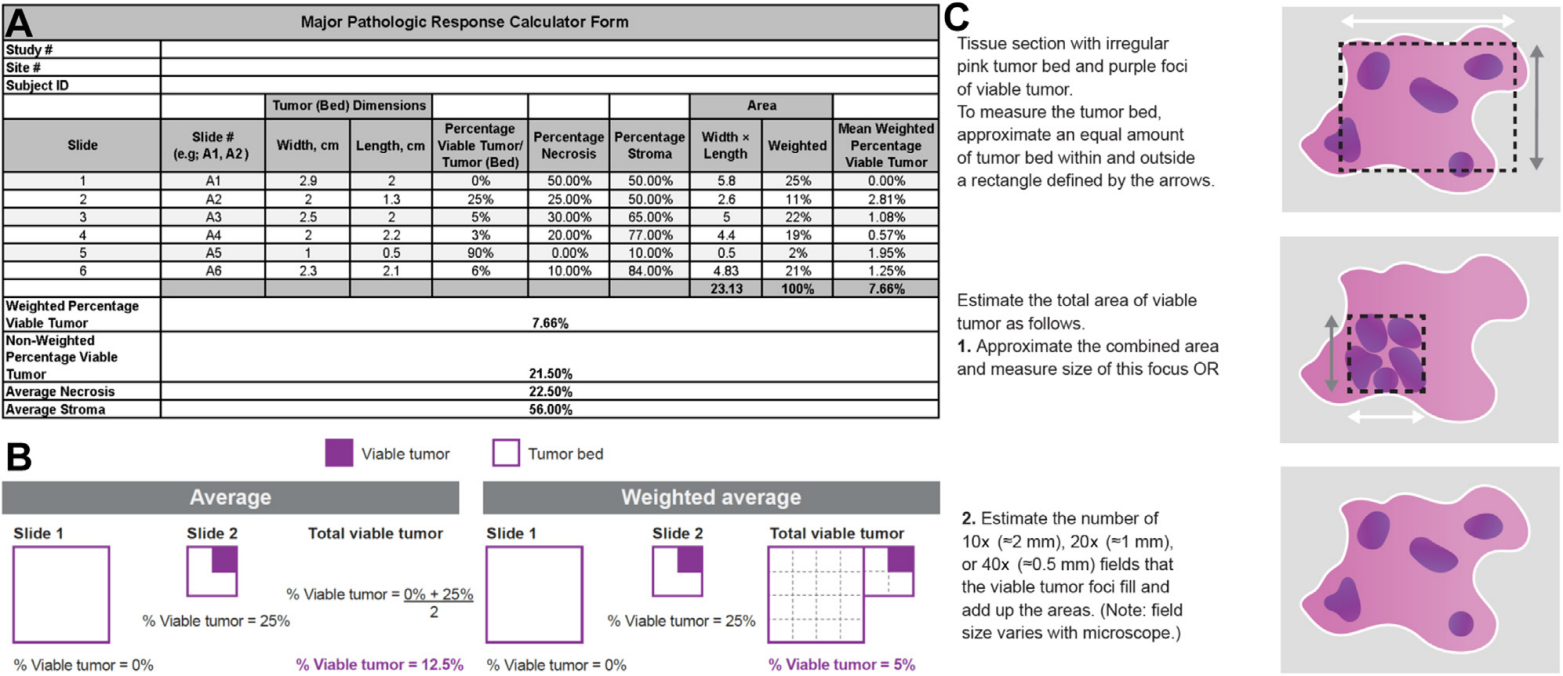
Using the MPRCT in microscopic assessment of pathologic response. (*A*) Example of MPRCT data collection form. Tumor bed = viable tumor + necrosis + stroma. Default of individual percentage stroma is 100%; the actual value is displayed after values are entered for percentage viable tumor and percentage necrosis. (*B*) Schematic revealing differences in obtaining the unweighted (21.50%) and weighted (7.66%) average MPRs. (*C*) How to determine the length and width of viable tumor (purple foci) in the tumor bed (pink). White outline borders an irregularly shaped tumor bed. The black dashed rectangle provides the best-fitted regular shape to assess width (white arrow) and height (gray arrow). #, number; ID, identification; MPR, major pathologic response; MPRCT, major pathologic response calculator tool.

The MPRCT determines MPR, as defined by Pataer et al.,^14^ and captures as percentages the following three elements of the “treatment effect in primary tumor” outlined in the recommended IASLC synoptic template: (1) viable tumor, (2) necrosis, and (3) stroma (which includes fibrosis and inflammation).^10^ The MPRCT has designated fields for entering the dimensions (i.e., width and length) of the tumor bed and percentage of viable tumor and necrosis on each slide (Fig. 1*A*). The stroma percentage is automatically computed on the basis of the following formula: % viable tumor + % necrosis + % stroma = 100%. For each entry, pre-embedded formulas calculate the area of mean viable tumor (both weighted and unweighted), necrosis, and stroma. The weighted average takes into account the varying proportions of viable tumor across tissue sections (Fig. 1*B*).

The MPRCT has safeguards to detect errors (e.g., cells with missing data) and prevent inadvertent mathematical miscalculations, such as a mean or sum greater than 100% for viable tumor plus necrosis plus stroma on each slide. For the computed total tumor bed area, the MPRCT can establish whether the entered values are within or outside an expected range for the specified gross dimensions.

In addition to standardization, functionality, and ease of use, the MPRCT can be used to provide data for evidence-based determination of optimal sampling and microscopic assessment strategies and for the discovery of new potential surrogate end points.

## MPRCT and Microscopic Assessment

The contribution of each tumor bed component (viable tumor, necrosis, and stroma) to MPR as a surrogate end point and the required granularity of documentation remain to be determined. Although the IASLC guidelines recommend recording tumor proportions in 10% increments (or single digits if <5%),^10^ the MPRCT permits the entry of any whole number between 0 and 100 and allows a more accurate calculation of the tumor proportion. Comparison of outcomes using the proportions captured as a continuous variable in the MPRCT with those obtained by rounding to the nearest 10% may provide an indication of the depth of stringency required.

The MPRCT requires the entry of tumor length and width for each tumor bed slide (Fig. 1*B*). Although not substitutes for precise digital annotation, width and length measured using a ruler, ocular micrometer, or known field diameter (Fig. 1*C*) increase objectivity compared with the “eyeball” method,^10^ are adoptable by all laboratories, and provide a more uniform and standardized system for tumor bed analysis. Measuring the width and length permits the calculation of the weighted percentage of mean viable tumor to allow for different proportions of tumor bed on each slide (Fig. 1*A* and *B*). The weighted percentage of the mean viable tumor for each patient can be calculated as: $\overset{n}{\underset{i=1}{\sum }}\frac{l_{i}w_{i}}{T}V_{i}$, where *n* is the number of slides, *l*_*i*_ is the length of the tumor bed under evaluation in slide *i*, w_*i*_ is the width of the tumor bed under evaluation in slide *i*, *T* is the total area of all slides for the patient (i.e., $T=\;\overset{n}{\underset{i=1}{\sum }}l_{i}w_{i}$), and *V*_*i*_ is the percentage of slide *i* comprising viable cells.

The MPRCT also allows calculation of the unweighted percentage mean viable tumor, as described previously.^14^ Whether the distinction between weighted and unweighted average percentage viable tumor is consequential or relevant only in instances where the percentage of mean viable tumor is similar to the MPR cutoff requires further investigation. Furthermore, the MPRCT allows calculation of the mean percentage of necrosis and stroma to enable determination of their significance, if any. Nevertheless, the MPRCT does not have the facility to allow differentiation between fibrosis and inflammation in stroma, nor to facilitate the quantification or subclassification of inflammation or type of fibrosis.

## MPRCT and Tumor Sampling

Recent clinical trials of neoadjuvant checkpoint inhibitors included patients with stages I to IIIA NSCLC.^6^^,^^8^^,^^23^ Although potentially resectable, a stage IIIA NSCLC tumor may exceed 7 cm in diameter. The sampling methodology described for previous studies has been inconsistent (Table 1). The IASLC recommends complete examination of tumors less than or equal to 3 cm and selective sampling of sections of larger tumors, with at least one section taken per centimeter of greatest tumor diameter.^10^ For tumors more than 3 cm in diameter, the cross-section found by gross inspection to be the most representative of the entire tumor (and tumor bed if identifiable grossly) should be sampled.

**Figure 2:**
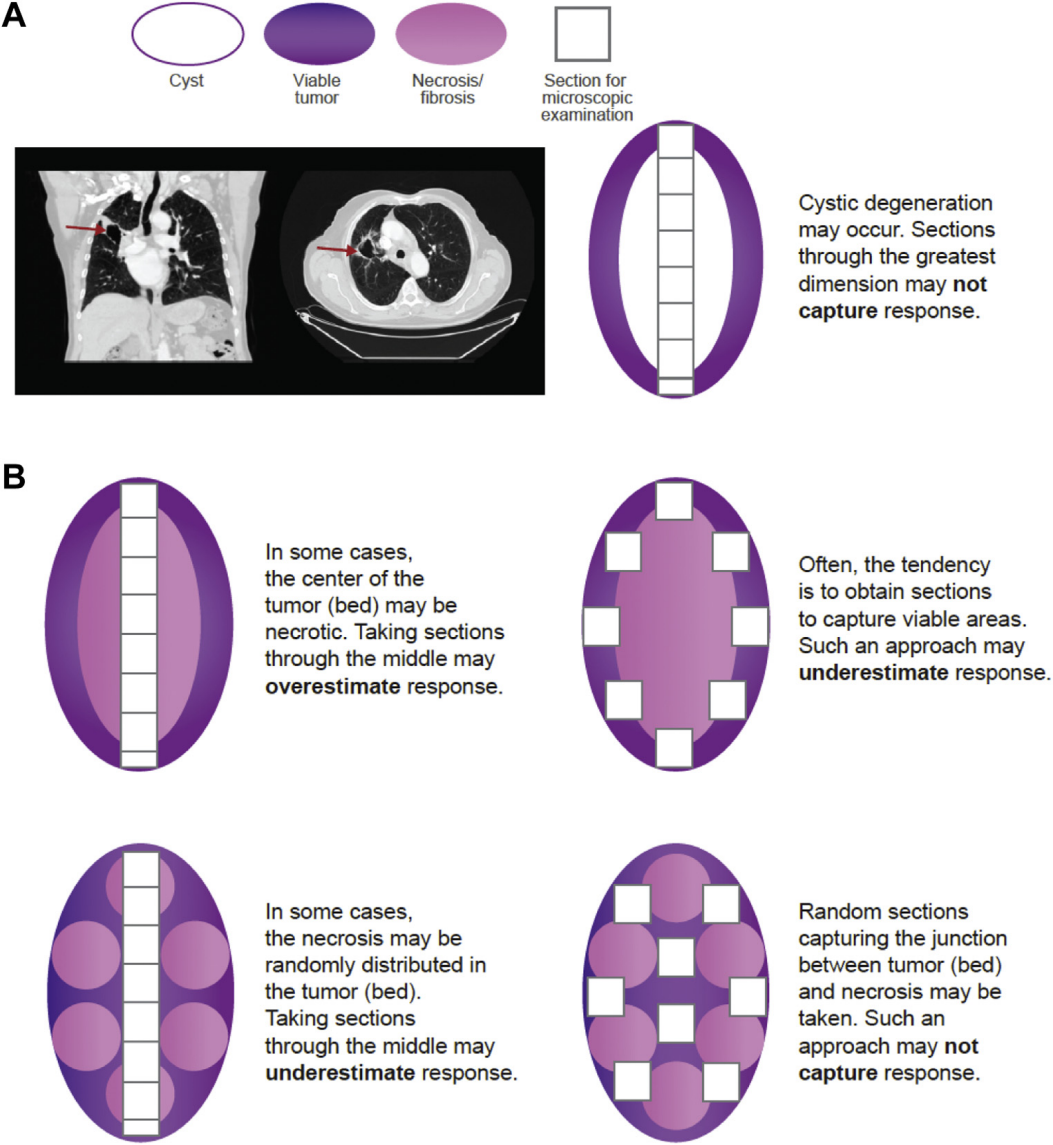
Sampling for microscopic examination under different scenarios. (*A*) Cystic cavitation after neoadjuvant treatment (indicated by red arrows on the scan) may not be captured in the denominator of tumor bed size. This is considered a challenging scenario. (*B*) Heterogeneity in response and sampling can influence the calculated percentage of mean viable tumor.

Although focused and partial tumor bed sampling are sufficient for routine pathologic staging and reporting, their relative utility in relation to the reproducibility and accuracy of MPR derived from these remains unproven. Heterogeneous responses to neoadjuvant treatment have been described.^14^ Other than a greater propensity for residual tumor along the lesion periphery reported in one study,^4^ the distribution—central, peripheral, or random—of cystic degeneration, viable cells and necrosis (Fig. 2*A* and *B*) in the tumor beds has not been formally evaluated. Heterogeneity in both response and sampling (Fig. 2*B*) can influence the calculated percentage of mean viable tumor. An enhanced understanding of response heterogeneity requires adequate tumor bed evaluation. Determining the optimal proportion of tumor to be sampled entails striking a balance between partial versus complete sampling, without placing unwarranted burden on pathologists and laboratories (Tables 1 and 2 and Supplementary Table 1).^24^^,^^25^

**Table 2:** Proposed Strategy for Tumor Bed Sampling and Microscopic Assessment (Including in Challenging Scenarios) in Conjunction With the MPRCT

Sampling Methodology		Microscopic Assessment for Calculation of Pathologic Response
Tumor Diameter, cm	Percentage of Tumor (Bed) for Microscopic Assessment	
≤3 cm tumor (bed)	Submit entire tumor (bed) (100%)	• Accurate calculation of proportions of viable tumor area, necrosis, and stroma based on their dimensions on each slide, which are entered into the MPRCT to calculate the weighted percentage of mean viable tumor (see Fig. 1)
>3 cm tumor (bed) without pCR	≥50% of tumor (bed)	• Pathologic assessment of necrosis and stroma – Characterizing extracellular mucin as tumor or stroma: rare neoplastic cells in otherwise identical and adjacent extracellular mucin pools represent residual tumor and stroma, respectively – Defining regression bed: use IASLC definition of stroma; viable nontumor tissue/cells are counted and a distinction between native and regressive stroma is not attempted – Assessing cystic changes: further investigation, because empty cystic cavities do not meet the criteria of viable tumor, stroma, or necrosis; because they are rare and not tumor necrosis or stroma, cysts are not included in the MPRCT – Hilar tumors: 1. MPR or pCR: examine microscopic tumor bed features for vessels with myointimal thickening with an undulating elastica, expanded adventitia around blood vessels and accompanying inflammation and fibroplasia, and absence of orderly alignment of collagen, all of which indicate tumor bed 2. Significant residual tumor: neoplastic cells extending directly into the lymph node are considered viable tumor bed
Tumor any size with pCR	Submit entire tumor (bed) (100%)	• MPRCT allows the use of unrestricted continuous values (vs. nearest “eyeballed” multiples of 10%)

IASLC, International Association for the Study of Lung Cancer; MPR, major pathologic response; MPRCT, major pathologic response calculator tool; pCR, pathologic complete response.

**Figure 3:**
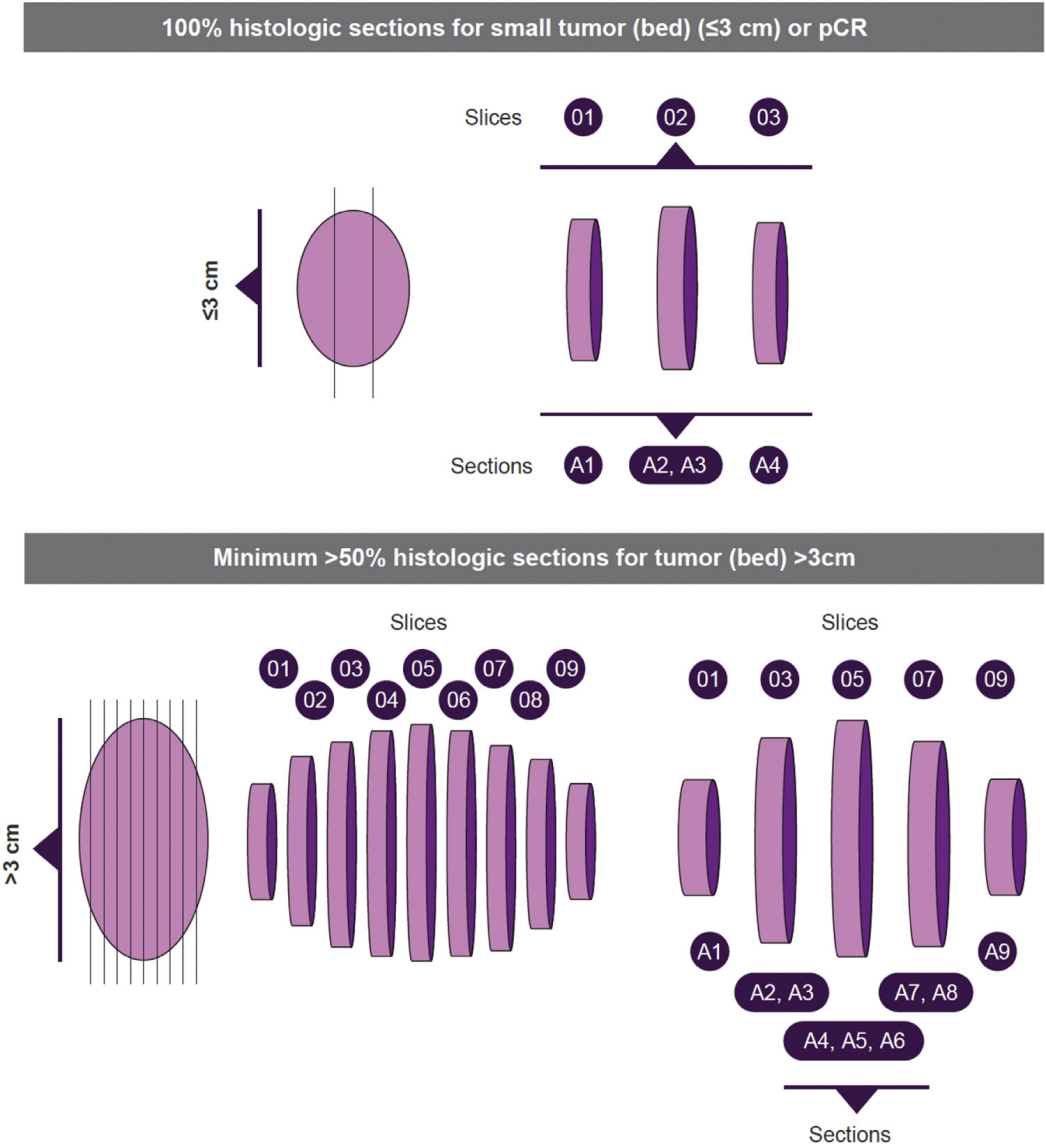
Recommended minimum number of tumor (bed) sections to be submitted and strategy for documentation. pCR, pathologic complete response.

The proposed strategy for tumor bed sampling and microscopic assessment therefore differs from the recommendations of the IASLC (summarized in Table 1) in some aspects. We propose examination of the entire tumor bed when the diameter is less than or equal to 3 cm or where pCR has occurred (Table 2). In the absence of evidence-based data for tumors more than 3 cm, we propose the examination of a minimum of 50% of the tumor (and tumor bed if grossly identifiable) sampled from alternate sections (Fig. 3). This captures heterogeneity and removes bias introduced during standard pathology practice—selection of viable areas and avoidance of necrotic regions—that can underestimate response. By contrast, the IASLC guidelines recommend sampling of regions with greatest viable tumor.^10^ Our proposed method also allows assessment of similar proportions of tumor beds that are more than 3 cm, whereas the standard pathology approach (1 section per centimeter of greatest tumor diameter) results in sampling of progressively diminishing proportions as the tumor size increases (Supplementary Table 1). For example, using the standard approach, 100% of a 3 cm-diameter tumor bed is examined, compared with only 6% of a 7 cm-diameter tumor bed. In the absence of viable tumor in the initial sections, the remaining tumor bed should be completely examined to confirm pCR. Our preliminary observations suggest that this is not an overly onerous recommendation; this will become even less time consuming in the future with the adoption of digital pathology and artificial intelligence (AI). Preliminary data have revealed high concordance between pathologist-assessed and AI-powered digital assessment of MPR.^26^

## Balancing Advantages and Limitations of the Proposed Methodology

Compared with the “eyeball” estimation method mentioned in the IASLC guidelines, measuring viable tumor length and width increases the time needed for assessment. This is compounded by our proposal to evaluate more than or equal to 50% of a tumor bed greater than 3 cm in diameter. Nevertheless, submission of more than or equal to 50% of the tumor bed may likely mitigate interobserver variability by providing more data points to normalize discrepancies and outliers. For example, evidence may reveal that for tumor beds greater than 3 cm, one section per centimeter at the greatest tumor bed diameter is sufficient. Alternatively, such tumor beds may require sampling of more than or equal to 50%, but detailed microscopic assessment of only a subset of slides (e.g., abundant viable tumor on the initial slides is unlikely to be offset by an absence of tumor in the remaining slides and reach MPR). Currently, no evidence-based studies investigating the ideal number of sections or percentage of tumor examined are available, although others in the field have also recommended submission of the entire tumor bed to provide robust data for informed decision-making.^24^

Ultimately, the proposed sampling strategy may exceed what is necessary to calculate MPR accurately. Until more data become available, this unbiased approach and data from the MPRCT can be used in formal analyses to determine the optimum sampling strategy that balances sensitivity against overuse of resources. Similarly, examination of the entire tumor bed to confirm pCR may seem excessive, but the ability to correlate accurately confirmed pCR with improved outcomes would provide robust evidence for pCR as a surrogate end point of survival, similar to that established for high-risk *HER2*-positive early-stage breast cancer.^27^

## MPRCT and Interobserver Agreement

Few studies dedicated to interobserver MPR variability have been published, but limited data on pathologic reproducibility are available.^4^^,^^8^^,^^19^^,^^28^ In studies of neoadjuvant NSCLC chemoradiotherapy^4^ and chemoimmunotherapy,^8^ no differences in pathology findings were found between different study centers; however, “agreement” is not defined for either study. In their evaluation of NSCLC postneoadjuvant chemotherapy, Qu et al.^19^ described high interobserver reproducibility between two experienced pathologists using increments of 5% to record viable tumor and intraclass correlation for statistical analysis. These investigators defined the threshold of acceptable reproducibility for viable tumor proportion as the differences between measurements from two pathologists being within the limits of agreement approximately 95% of the time.^19^ In another small study of neoadjuvant nivolumab, a median 5% variability in histopathologic features (range: 0%–29%) was reported for each sample reviewed by four pathologists.^28^ In a retrospective study that evaluated agreement regarding MPR assessment between two pathologists (one trained, one untrained), levels of agreement were high after in-person training by the experienced pathologist (*R*^2^ = 0.994).^12^

Although these data are encouraging, larger studies are needed to validate levels of concordance among general and thoracic pathologists and to define an acceptable range of discordance. Data from the MPRCT can be used to develop the best methodology for achieving high interobserver agreement and correlation with outcomes (e.g., unrestricted continuous values can be compared with those rounded to nearest multiples of 10). Conceivably, the methodology used might vary by case: unrestricted continuous values might be used if the percentage of viable tumor is near the 10% cutoff for MPR, and rounded values might be used in other scenarios. In addition, the MPRCT data can be used to identify cases that have data points revealing significant discrepancies for adjudication. It can also be used to identify trends and biases among pathologists (e.g., whether a given pathologist averages higher estimates) and sharpen criteria for the parameters assessed (e.g., exactly what constitutes stroma to the individual assessor). The details provided by the MPRCT permit granular analysis at a case or slide level (e.g., agreement among pathologists on slides that contain tumor bed).

## Challenging Scenarios in Pathologic Assessment

Although generally simple and reproducible, distinguishing between tumor or tumor bed and stroma can sometimes be challenging in the context of MPR assessment, and there are a number of situations in which identification of stroma becomes problematic. We propose a methodology to help overcome these challenges (Table 2), but correlation with clinical outcomes will be required to determine whether these proposals should be broadly adopted or modified further. The subsequent examples highlight common challenges, but overall, they represent a small minority of cases.

### Is Extracellular Mucin “Tumor” or “Stroma”?

It is unclear whether extracellular mucin of invasive mucinous adenocarcinomas should be considered viable tumor or stroma. On the basis of the IASLC guidelines, mucin pools represent either viable tumor or stroma, depending on the presence or absence, respectively, of accompanying viable neoplastic cells.^10^ Hence, otherwise identical extracellular mucin pools may be considered either residual tumor or stroma. We favor interpreting all extracellular mucin as stroma (Table 2). Although this approach may also be extrapolated to “colloid carcinoma,” this rare subtype has not been encountered in our observations.

### Do the Cores of Papillary Tumor Represent “Tumor” or “Stroma”?

The appropriate categorization of fibrovascular cores (a combination of fibrosis, capillaries, and lymphoid cells) as viable tumor or stroma remains debatable. These are a prominent and defining trait of invasive papillary predominant adenocarcinomas. On the basis of the IASLC definition,^10^ fibrovascular cores are interpreted as stroma. This approach minimizes well-known interobserver variability in papillary versus nonpapillary assignment,^29^ provides consistency across all histologic subtypes, increases reproducibility, and obviates subjective distinction between innate features and response.

### What Constitutes the “Regression Bed”?

The term “regression bed” has been used for stroma and is characterized by immune activation, massive tumor cell death, and tissue repair (e.g., lymphocytes, macrophages, cholesterol clefts, and proliferative fibrosis).^28^ The IASLC definition of stroma is more lenient and does not require the presence of regressive changes,^10^ with the justification that they may be indistinguishable from native tumor characteristics. For example, central fibrosis of treatment-naive adenocarcinomas may overlap morphologically with proliferative fibrosis of a regression bed. Both perspectives are valid and defensible.

The IASLC definition for all NSCLC nonmucinous subtypes has been adopted: viable cells are counted and a distinction between native and regressive stroma is not attempted. Evaluating only viable tumor cells is less complex, more objective, and therefore presumably more reproducible. Furthermore, this method may lend itself better to digital analysis and AI; distinguishing tumor cells from stroma is easier than distinguishing native stroma from regressive stroma.

### Assessing Cases With Cystic Change in the Tumor Bed

Another challenge in MPR assessment is that after neoadjuvant therapy, some NSCLC tumors degenerate, resulting in an empty cystic cavity or cavities (Fig. 2*A*). In these situations, the tumor bed size (representing the denominator) may encompass or omit the empty space, and the choice of which option to use as a denominator will significantly affect the calculated percentage of mean viable tumor. This infrequent scenario requires further investigation and correlation with imaging, because the empty spaces do not meet the morphologic criteria of viable tumor, stroma, or necrosis. In line with this practice, other sizable empty spaces (e.g., bronchial lumen) are subtracted from the tumor bed in hilar tumors. As before, this assumption may be more suited to digital analysis.

### Assessing Response in Hilar Tumors

Evaluation of hilar tumors is challenging in two situations. In the first, where extensive response (MPR or pCR) is present, macroscopic and microscopic identification of the tumor bed is difficult owing to the minimal and subtle distinguishing features of the “regression bed” relative to the adventitia of large vessels and airways. Microscopic tumor bed features seem to accentuate and expand the adventitia around the blood vessels and large airways, often with accompanying inflammation and fibroplasia, and these are useful in confirming and localizing the tumor bed. After neoadjuvant treatment, the vessels in the tumor bed were found to have myointimal thickening with an undulating elastica, whereas in nontumor bed areas, they are unadulterated. The native adventitia around the airways may merge seamlessly with therapy-related fibrosis; an absence of parallel and orderly alignment of collagen is indicative of a tumor bed.

In the second situation encountered with hilar tumors, significant residual tumor may be present. The distinctions between tumor bed, direct extension of the tumor into the lymph nodes, and lymph node metastasis are obscured, particularly when tumors are associated with significant inflammation. Although categorized as N1 for staging, for MPR assessment,^10^ the neoplastic cells extending directly into the lymph node are considered viable tumor bed. The presence of a well-defined and intact capsule is used to facilitate the distinction of a lymph node with metastasis from direct tumor extension.

Additional instances in which the distinction between tumor bed and nontumor bed can be unclear include postobstructive changes and underlying fibrotic interstitial lung disease (that may not have been clinically recognized). An initial and rapid review of all the slides provides an informative overview of the spectrum of changes in such cases.

## Future Directions for the MPRCT

The MPRCT and these proposed sampling strategies are being used to collect data systematically during neoadjuvant therapy clinical trials to determine the ideal sampling method to calculate MPR accurately while balancing sensitivity against excessive use of resources. At present, neither the IASLC recommendations nor any other study has provided sufficient evidence-based data to indicate which method is optimal. Data from ongoing Phase 3 neoadjuvant trials including IMpower030 and from other studies using this MPRCT will shed further light on this, once available.

Comprehensive data collection for the MPRCT places the onus on pathologists but is likely to yield high returns on the initial time investment. Readily accessible and detailed information obtained from the outset improves efficiency by obviating microscopic re-review to address inquiries from regulatory agencies. It also enables testing of new hypotheses and the exploration of additional end points, especially for phase 3 clinical trials with hopes of ultimately establishing a surrogate end point and investigating a new class of drugs, such as immunotherapies in the neoadjuvant setting. Most of these tasks can be accomplished by examining the raw MPRCT data, extrapolating data (e.g., rounding recorded percentages to nearest multiples of 10, as described previously), or performing multivariate analysis using information from synoptic reports of NSCLC resections, predictive and prognostic ancillary studies, and radiologic findings, among others.

The value of these types of analyses is illustrated by findings from two contemporary studies. In a clinical trial of chemoimmunotherapy, more patients with squamous cell carcinoma than adenocarcinomas had MPR and pCR.^6^ In another study, assessment of resected NSCLC specimens after neoadjuvant chemotherapy revealed that less than or equal to 10% viable tumor, the widely adopted definition of MPR, was applicable to squamous cell carcinomas but not adenocarcinomas, for which the optimal threshold was 65%.^19^ As suggested by Qu et al.,^19^ this finding has significant implications for future clinical trials, and additional studies are needed to substantiate the results and confirm the value of stratifying lung adenocarcinomas by predominant subtype.

The differences between our proposed sampling and assessment methodology (Table 2) and the IASLC recommendations (Table 1) may result in more accurate and effective data capture in areas of heterogeneity and may reduce the bias introduced by standard pathology practice (e.g., selecting viable areas and avoiding necrotic regions) that can lead to underestimation of pathologic response. Clinical and pathologic data from the ongoing IMpower030 study will confirm whether our proposed methodology will increase the accuracy of the calculated MPR while maintaining the balance between sensitivity and overuse of resources. Further analyses using the MPRCT will inform the adoption of a generic, uniform, or tailored approach to assessment of pathologic response that is dependent on specific parameters (e.g., tumor bed size, NSCLC subtype, treatment modality). With readily available and encompassing data from the outset, these and other queries can be expeditiously addressed.

The proposed methodology is described for use in clinical trials; how it could be adopted in routine clinical practice remains to be determined. If the prognostic significance of pathologic response to neoadjuvant therapy for breast cancer^30^ is a harbinger, then it is likely that clinicians in routine practice will want MPR data on patients with NSCLC once the usefulness is confirmed. Given such a development, comparisons of MPR assessments between local sites and central pathology laboratories will provide additional data on reproducibility, the impact of residual viable tumor on clinical management, and future use in real-world data studies and nontrial clinical cases.

In conclusion, collection of comprehensive data in a standardized format using the MPRCT will facilitate the validation of protocols that include pCR and MPR end points with the aim of establishing them as surrogate end points of neoadjuvant treatment efficacy while also providing insight into areas that require further investigation.

## CRediT Authorship Contribution Statement

**Anjali Saqi:** Conceptualization, Methodology, Validation, Writing - original draft, Writing - review & editing, Visualization, Supervision, Project administration.

**Kevin O. Leslie, Andre L. Moreira, Sylvie Lantuejoul, Catherine Ann Shu, Naiyer A. Rizvi, Joshua R. Sonett:** Writing - original draft, Writing - review & editing, Visualization.

**Kosei Tajima:** Methodology, Validation, Writing - original draft, Writing - review & editing, Visualization.

**Shawn W. Sun, Barbara J. Gitlitz:** Conceptualization, Validation, Writing - original draft, Writing - review & editing, Visualization, Supervision, Project administration.

**Thomas V. Colby**: Conceptualization, Writing - original draft, Writing - review & editing, Supervision.

## References

[1] Sung H., Ferlay J., Siegel R.L. (2021). Global cancer statistics 2020: GLOBOCAN estimates of incidence and mortality worldwide for 36 cancers in 185 countries. CA Cancer J Clin.

[2] Siegel R.L., Miller K.D., Fuchs H.E., Jemal A. (2021). Cancer statistics, 2021. CA Cancer J Clin.

[3] Poettgen C., Theegarten D., Eberhardt W. (2007). Correlation of PET/CT findings and histopathology after neoadjuvant therapy in non-small cell lung cancer. Oncology.

[4] Blaauwgeers J.L., Kappers I., Klomp H.M. (2013). Complete pathological response is predictive for clinical outcome after tri-modality therapy for carcinomas of the superior pulmonary sulcus. Virchows Arch.

[5] Rusch V.W., Giroux D.J., Kraut M.J. (2007). Induction chemoradiation and surgical resection for superior sulcus non-small-cell lung carcinomas: long-term results of Southwest Oncology Group Trial 9416 (Intergroup Trial 0160). J Clin Oncol.

[6] Shu C.A., Gainor J.F., Awad M.M. (2020). Neoadjuvant atezolizumab and chemotherapy in patients with resectable non-small-cell lung cancer: an open-label, multicentre, single-arm, phase 2 trial. Lancet Oncol.

[7] Rothschild S.I., Zippelius A., Eboulet E.I. (2021). SAKK 16/14: durvalumab in addition to neoadjuvant chemotherapy in patients with stage IIIA(N2) non-small-cell lung cancer-a multicenter single-arm phase II trial. J Clin Oncol.

[8] Provencio M., Nadal E., Insa A. (2020). Neoadjuvant chemotherapy and nivolumab in resectable non-small-cell lung cancer (NADIM): an open-label, multicentre, single-arm, phase 2 trial. Lancet Oncol.

[9] William W.N., Pataer A., Kalhor N. (2013). Computed tomography RECIST assessment of histopathologic response and prediction of survival in patients with resectable non-small-cell lung cancer after neoadjuvant chemotherapy. J Thorac Oncol.

[10] Travis W.D., Dacic S., Wistuba I. (2020). IASLC multidisciplinary recommendations for pathologic assessment of lung cancer resection specimens after neoadjuvant therapy. J Thorac Oncol.

[11] Cascone T., William W.N., Weissferdt A. (2021). Neoadjuvant nivolumab or nivolumab plus ipilimumab in operable non-small cell lung cancer: the phase 2 randomized NEOSTAR trial. Nat Med.

[12] Weissferdt A., Pataer A., Vaporciyan A.A. (2020). Agreement on major pathological response in NSCLC patients receiving neoadjuvant chemotherapy. Clin Lung Cancer.

[13] Junker K., Thomas M., Schulmann K., Klinke F., Bosse U., Müller K.M. (1997). Tumour regression in non-small-cell lung cancer following neoadjuvant therapy. Histological assessment. J Cancer Res Clin Oncol.

[14] Pataer A., Kalhor N., Correa A.M. (2012). Histopathologic response criteria predict survival of patients with resected lung cancer after neoadjuvant chemotherapy. J Thorac Oncol.

[15] Pataer A., Weissferdt A., Vaporciyan A.A. (2021). Evaluation of pathologic response in lymph nodes of patients with lung cancer receiving neoadjuvant chemotherapy. J Thorac Oncol.

[16] Betticher D.C., Hsu Schmitz S.F., Totsch M. (2003). Mediastinal lymph node clearance after docetaxel-cisplatin neoadjuvant chemotherapy is prognostic of survival in patients with stage IIIA pN2 non-small-cell lung cancer: a multicenter phase II trial. J Clin Oncol.

[17] Chaft J.E., Rusch V., Ginsberg M.S. (2013). Phase II trial of neoadjuvant bevacizumab plus chemotherapy and adjuvant bevacizumab in patients with resectable nonsquamous non-small-cell lung cancers. J Thorac Oncol.

[18] Hellmann M.D., Chaft J.E., William W.N. (2014). Pathological response after neoadjuvant chemotherapy in resectable non-small-cell lung cancers: proposal for the use of major pathological response as a surrogate endpoint. Lancet Oncol.

[19] Qu Y., Emoto K., Eguchi T. (2019). Pathologic assessment after neoadjuvant chemotherapy for NSCLC: importance and implications of distinguishing adenocarcinoma from squamous cell carcinoma. J Thorac Oncol.

[20] Chui M.H., Kandel R.A., Wong M. (2016). Histopathologic features of prognostic significance in high-grade osteosarcoma. Arch Pathol Lab Med.

[21] Guarneri V., Broglio K., Kau S.W. (2006). Prognostic value of pathologic complete response after primary chemotherapy in relation to hormone receptor status and other factors. J Clin Oncol.

[22] Thomas J.S.J., Provenzano E., Hiller L. (2017). Central pathology review with two-stage quality assurance for pathological response after neoadjuvant chemotherapy in the ARTemis Trial. Mod Pathol.

[23] Forde P.M., Chaft J.E., Smith K.N. (2018). Neoadjuvant PD-1 blockade in resectable lung cancer. N Engl J Med.

[24] Oramas D.M., Moran C.A. (2021). Major pathologic response in patients treated for non-small cell carcinoma of the lung: is there a magic number in the histologic sections to be evaluated?. Adv Anat Pathol.

[25] Weissferdt A., Pataer A., Swisher S.G. (2021). Controversies and challenges in the pathologic examination of lung resection specimens after neoadjuvant treatment. Lung Cancer.

[26] Dacic S., Travis W.D., Giltnane J.M. (2021). Artificial intelligence (AI)–powered pathologic response (PathR) assessment of resection specimens after neoadjuvant atezolizumab in patients with non-small cell lung cancer: results from the LCMC3 study. J Clin Oncol.

[27] US Department of Health and Human Services, Food and Drug Administration, Oncology Center of Excellence, Center for Drug Evaluation and Research, Center for Biologics Evaluation and Research Pathological complete response in neoadjuvant treatment of high-risk early-stage breast cancer: use as an endpoint to support accelerated approval guidance for industry. https://www.fda.gov/media/83507/download.

[28] Cottrell T.R., Thompson E.D., Forde P.M. (2018). Pathologic features of response to neoadjuvant anti-PD-1 in resected non-small-cell lung carcinoma: a proposal for quantitative immune-related pathologic response criteria (irPRC). Ann Oncol.

[29] Thunnissen E., Beasley M.B., Borczuk A.C. (2012). Reproducibility of histopathological subtypes and invasion in pulmonary adenocarcinoma. An international interobserver study. Mod Pathol.

[30] Spring L.M., Fell G., Arfe A. (2020). Pathologic complete response after neoadjuvant chemotherapy and impact on breast cancer recurrence and survival: a comprehensive meta-analysis. Clin Cancer Res.

[31] Forde P.M., Spicer J., Lu S. (2021). Nivolumab + platinum doublet chemotherapy vs chemotherapy as neoadjuvant treatment for resectable (IB IIIA) non small cell lung cancer in the phase 3 CheckMate 816 trial. Cancer Res.

