# Supplementary material for: Assessing Pathologic Response in Resected Lung Cancers: Current Standards, Proposal for a Novel Pathologic Response Calculator Tool, and Challenges in Practice
Source: JTO Clin Res Rep. 2022 Mar 19;3(5):100310. doi: 10.1016/j.jtocrr.2022.100310 (PMC9044000; doi:10.1016/j.jtocrr.2022.100310)
Supplement: Supplemental Table 1 [file mmc1.docx]

**Supplemental Table 1**. Approximate number of sections and percentage of total volume that would be evaluated for tumors (or tumor beds) ranging from 3 to 7 cm in diameter when submitted entirely (100%) and at 1 cassette per centimeter. Note the substantial reduction in percentage of total tumor evaluated when submitting 1 cassette per centimeter: 6% for a 7 cm tumor (bed) compared with 100% for a 3 cm tumor (bed).

| **Greatest Diameter of Tumor (Bed), cm** | **Volume of Tumor (Bed), cm^3a^** | **No. of Sections Evaluated When Submitting 100%^b^** | **No. of Sections Evaluated When Submitting 1 per cm** | **Volume of Tumor (Bed) Evaluated When Submitting 1 per cm, cm^3^** | **Percentage of Tumor (Bed) Evaluated When Submitting 1 per cm, %** |
| --- | --- | --- | --- | --- | --- |
| 3 | 14.14 | 9 | N/A^c^ | N/A (14.14 total)^c^ | N/A (100)^c^ |
| 4 | 33.51 | 21 | 4 | 6.4 | 19 |
| 5 | 65.45 | 41 | 5 | 8 | 12 |
| 6 | 113.10 | 71 | 6 | 9.6 | 8 |
| 7 | 179.59 | 112 | 7 | 11.2 | 6 |

NA, not applicable.

^a^For these calculations, a spherical tumor (bed) shape was assumed

^b^Section volume is assumed to be 0.4 cm × 2 cm × 2 cm = 1.6 cm^3^

^c^For tumor (beds) ≤3 cm in diameter, the entire tumor is evaluated
